# Association between Genetic Variants and Diabetes Mellitus in Iranian Populations: A Systematic Review of Observational Studies

**DOI:** 10.1155/2015/585917

**Published:** 2015-10-26

**Authors:** Mehrnoosh Khodaeian, Samaneh Enayati, Ozra Tabatabaei-Malazy, Mahsa M. Amoli

**Affiliations:** ^1^Endocrinology and Metabolism Research Center, Endocrinology and Metabolism Clinical Sciences Institute, Tehran University of Medical Sciences, Tehran, Iran; ^2^Diabetes Research Center, Endocrinology and Metabolism Clinical Sciences Institute, Tehran University of Medical Sciences, Tehran, Iran

## Abstract

*Introduction.* Diabetes mellitus as the most prevalent metabolic disease is a multifactorial disease which is influenced by environmental and genetic factors. In this systematic review, we assessed the association between genetic variants and diabetes/its complications in studies with Iranian populations. *Methods.* Google Scholar, PubMed, Scopus, and Persian web databases were systematically searched up to January 2014. The search terms were “gene,” “polymorphism,” “diabetes,” and “diabetic complications”; nephropathy, retinopathy, neuropathy, foot ulcer, and CAD (coronary artery diseases); and Persian equivalents. Animal studies, letters to editor, and in vitro studies were excluded. *Results.* Out of overall 3029 eligible articles, 88 articles were included. We found significant association between CTLA-4, IL-18, VDR, TAP2, IL-12, and CD4 genes and T1DM, HNF*α* and MODY, haptoglobin, paraoxonase, leptin, TCF7L2, calreticulin, ER*α*, PPAR-*γ*2, CXCL5, calpain-10, IRS-1 and 2, GSTM1, KCNJ11, eNOS, VDR, INSR, ACE, apoA-I, apo E, adiponectin, PTPN1, CETP, AT1R, resistin, MMP-3, BChE K, AT2R, SUMO4, IL-10, VEGF, MTHFR, and GSTM1 with T2DM or its complications. *Discussion.* We found some controversial results due to heterogeneity in ethnicity and genetic background. We thought genome wide association studies on large number of samples will be helpful in identifying diabetes susceptible genes as an alternative to studying individual candidate genes in Iranian populations.

## 1. Introduction

Diabetes mellitus, as the most prevalent metabolic disorder, is characterized by chronic hyperglycemia due to defect in insulin secretion by beta cells of Langerhans islets or resistance against insulin action [1–3]. More than 300 million people are suffering from diabetes mellitus all over the world and studies show that population aging, changes in lifestyle and improvement in detection techniques are most important factors in increasing the numbers of cases [4]. The prevalence of type 2 diabetes mellitus (T2DM) varies in different populations from less than 6% in most populations to more than 50% in Pima Indians [5]. In 2013 it was reported that in Middle East region about 35 million people suffered from diabetes. The prevalence of diabetes has been estimated as 382 million people throughout the world while nearly 176 million of them seem to be still undiagnosed. It is predicted that this prevalence reaches to 592 million by 2035. Diabetes mellitus can also cause complications in most of organs: heart, eye, kidney, and nervous system which has resulted in high economic cost and burden [6]. Therefore, diagnosis of disease in early stages is very important.

A systematic review showed that between years 1996 and 2004 the prevalence of type 2 diabetes in Iran was 24% and the risk was 1.7% greater in women. According to this report the prevalence of T2DM in Iran seems to be highest amongst developing countries. Previous reports on total urban population of Middle Eastern countries show the prevalence of T2DM as 3.4% in Sudan, 20% in United Arab Emirates, 8.5% in Bahrain, and 12.1% in India [7].

Diabetes Mellitus is categorized into the following groups.

Type 1 diabetes mellitus (T1DM) includes 5–10% of diabetic patients. Cellular-mediated autoimmune destruction of the beta-cells of the pancreas results in T1DM. It classically occurs in juveniles and affected patients are dependent on insulin injection in their lifetime and are very prone to ketosis [1, 8, 9].

T2DM includes 90–95% of patients with diabetes. Patients with type 2 diabetes may be asymptomatic for long period of time. Vascular complications such as nephropathy, neuropathy, retinopathy, and cardiovascular disease may develop in these patients. The impact of genetic component appears to be stronger in T2DM compared to T1DM [1, 8, 9].

Gestational diabetes mellitus (GDM) is another type which is observed during pregnancy and the prevalence may range from 1 to 14% in all pregnancies [1, 8, 9].

MODY (maturity onset diabetes of young) is monogenic form of diabetes comprised of several types with various features which is consisting of 1–5% of patients diagnosed as T2DM. The onset of this type of diabetes is normally before 25 years and its treatment is independent of insulin. MODY in its different forms is inherited in autosomal dominant pattern and presents as a result of mutation in transcription factors genes including* HNF4α* (hepatocyte nuclear factor),* HNF1β*,* IPF1* (insulin promoter factor), and* neuro-D1* [1, 8, 9].

There are also other types of diabetes which are considered as secondary to other conditions, for example, any damage to pancreas such as removal of pancreatic tissue, trauma, pancreatic carcinoma, and infection, or underlying diseases including endocrine diseases that alter different hormones secretion which are antagonist to insulin resulting in various clinical manifestations such as acromegaly, Cushing's syndrome, pheochromocytoma, glucagonoma, somatostatinoma, and diabetes. Diabetes (or carbohydrate intolerance) is also found in increased frequency with a large number of genetic syndromes such as Wolfram syndrome, which causes diabetes mellitus, diabetes insipidus, and other neurodegenerative disorders, MELAS (mitochondrial myopathy, encephalopathy, lactic acidosis, and stroke) which is presented with myopathy and encephalopathy caused by mitochondrial mutation, MIDD (maternally inherited diabetes and deafness) which causes diabetes, and IPEX (immune dysregulation, polyendocrinopathy, enteropathy, and X-linked syndrome) which is X-linked and alters immune system and causes multiple endocrine problems [1, 8, 9].

Diabetes mellitus is a multifactorial disease with both environmental and genetic causes affecting its presence and incidence. Genome wide association studies revealed the genetic heterogeneity of diabetes and the fact that difference in ethnicity can result in different susceptible genes associated with diabetes [10, 11]. Studies on candidate genes related to diabetes revealed that several genes including* PPRAG* (peroxisome proliferator-activates receptor gamma),* IRS1* and* 2* (insulin receptor substrate),* KCNJ11* (potassium inwardly rectifying channel), and* HNFA* are associated with T2DM. Genome wide association studies (GWAS) showed that many genes including Calpain 10 and TCF7L2 (transcription factor 7-like 2) are associated with T2DM.* HHEX* (hematopoietically expressed homeobox),* SLC30A8* (solute carrier family 30 (zinc transporter), member 8),* CDK2A/B* (cyclin-dependent kinase inhibitor 2A/B), and* IG2BP2* (insulin-like growth factor 2) are other genes which have been shown to be associated with T2DM based on GWAS. Some of these genes are expressed in beta cells or involved in insulin secretion pathways [12, 13]. Other candidate genes in association with T2DM include PPAR*γ*, ACE (angiotensin converting enzyme), MTHFR (methylene tetrahydrofolate reductase), FABP2 (fatty acid binding protein-2), and FTO (fat mass and obesity associated gene) [14]. GWAS was carried out in many populations including Finnish, French, and American Caucasians and showed different loci on most of the chromosomes associated with T2DM [15]. Systematic review and meta-analysis studies also confirmed the association of some genes such as PGC-1*α* (peroxisome proliferator-activated receptor gamma coactivator-1*α*) and adiponectin with T2DM [16, 17]. The genetic loci associated with T1DM have also been examined. For example, insulin (*INS*) gene VNTRs (variable number of tandem repeats) with a protective effect is a variants related to T1DM. In addition* PTPN22* (protein tyrosin phosphatase),* CTLA4*, and* IL2RA* genes have also been shown as candidates for T1DM due to their role in T-cell signaling. Studies have also confirmed the association between* IFIH1*,* CYP27B1*, and* CLEC16*, which are important in immune system and T1DM [18].

There is no inclusive information for genetic association studies of diabetes in Middle Eastern population including Iranian population. In order to make a comprehensive approach, we aimed to collectively investigate and gather data for association between genetic variants and type 2 diabetes in Iranian population in a systematic review study.

## 2. Research Design and Methods

This study is reported according to PRISMA (preferred reporting items for systematic reviews and meta-analyses) guideline [19].

### 2.1. Data Sources and Searches

We systematically searched international web databases: Google Scholar, PubMed, Scopus, and Persian web databases; IranMedex; and Magiran to investigate the association of genetic variants with diabetes and its complications in Iranian population up to January 2014. The search terms were “gene,” “polymorphism,” “diabetes,” “diabetes' complications,” nephropathy, retinopathy, neuropathy, foot ulcer, and CAD (coronary artery disease) and their MeSH terms and Persian equivalents with diabetes. At least three emails were sent to the corresponding author of articles which were not accessible as full text or had insufficient data. Duplicate articles and multiple publications from the same population were excluded and the most relevant data were used for the investigation. The references of all selected articles were investigated.

### 2.2. Study Selection

We included all observational population-based studies ≥100 sample size, which were conducted as case-control, cohort, or cross sectional. According to WHO criteria, diabetes mellitus was defined as FBS ≥ 7 mmol/L (126 mg/dL) or 2 h plasma glucose ≥11.1 mmol/L (200 mg/dL) [20]. In addition, we excluded animal studies, clinical trials, short communications, letters to editor, dissertations, in vitro studies, review articles, and population-based studies conducted in pregnant women. Two researchers, SE and MKH, independently reviewed title, abstract, and full text of each article to assess the eligible articles according to inclusion and exclusion criteria. Inclusion and exclusion of articles were supervised by a third reviewer (OTM) in case of conflicts. There was no limitation for language.

### 2.3. Data Extraction and Quality Assessment

The following data were extracted and presented in an excel sheet: author(s), year, genes and SNPs, patients' characteristics (sample size, age, and sex), city, study design, genotyping method, and significant association. Six selected items from the STROBE (strengthening the reporting of observational studies in epidemiology) checklist [21] was used for quality assessment of the included studies and assessed separately for each included article. The below items were considered: (a) clearly define the outcome and association between gene variants and diabetes; (b) give the eligibility criteria; (c) present key elements of study design; (d) report numbers or significance/nonsignificance statistically of outcome events; (e) give characteristics of study participants; and (f) describe the locations and relevant dates. All studies with quality score ≥3 were considered as high quality study and included in our systematic review.

### 2.4. Data Synthesis and Analysis

Due to heterogeneity in genotyping techniques and also differences in genetic variants studied in assessed included articles performing a meta-analysis was impossible.

## 3. Results

### 3.1. Search Results

**Figure 1:**
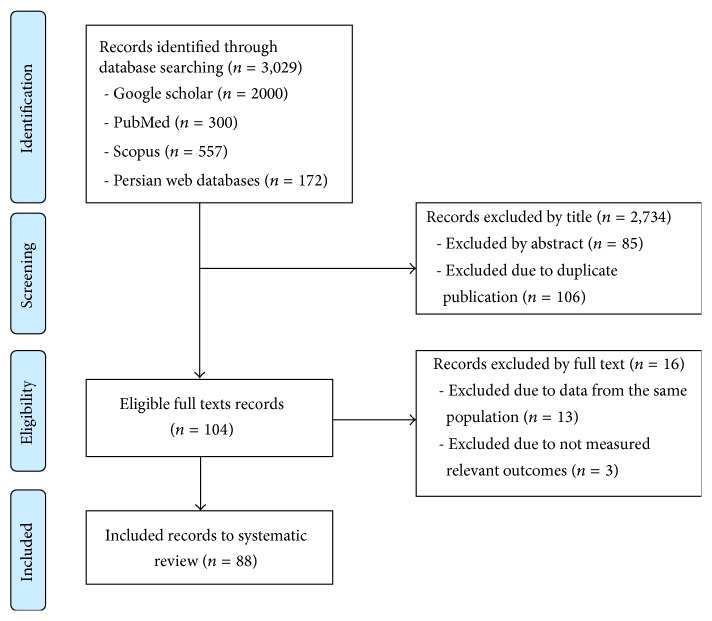
Flow diagram of study selection process.

A summary of the literature review process performed in this study is presented in a flow chart in Figure 1. In final step 88 eligible studies were included in this study and assessed due to the inclusion/exclusion criteria [22–109].  Table 1 shows the summary of each included investigation.

### 3.2. Studies Characteristics

Overall 27,396 diabetics and healthy subjects were studied in this systematic review. All of the subjects were recruited from total urban population. T2DM was the most investigated type of diabetes (42 studies) [22–75]. Among diabetes complications, diabetic nephropathy and CAD were the most examined, respectively [80–105]. Twelve studies were carried out on T1DM [22–33]. The least investigated complication was diabetic foot ulcer which was assessed only in one study [109]. Retinopathy and insulin resistance were also among the least investigated complications including 3 and 4 studies, respectively [76–79, 106–108]. Only one study assessed MODY in our review [34]. All of the studies were performed on both genders except for one which was performed on males [45].

Details of the included studies are described as below.

#### 3.2.1. T1DM Related Genes

All the studies in this subgroup including 12 studies [22–33] were in a case control study design using PCR (polymerase chain reaction) [33], PCR-RFLP (restriction fragment length polymorphism) [22, 23, 25, 26, 32], PCR-SSP (single specific primer) [24, 28–32], and ARMS-PCR (amplification refractory mutation system PCR) [27] method. In all studies both men and women were included and the overall 2519 subjects were under 40 years old.

Association of interleukins including IL-18 (interleukin) and IL-12 with T1DM revealed that the −137C/G polymorphism of IL-18 had significant association with T1DM (*P* = 0.037, *P* = 0.0001) while no significant relation was observed between −607A/C variant of this gene and T1DM [24, 28]. Investigating IL-12 also showed that there was a significant association between +1188A/C polymorphism of this gene and T1DM (*P* = 0.035) [30].

In 2 studies the association of +49A/G variant of CTLA-4 (cytotoxic T lymphocyte associated antigen 4) was investigated and it was revealed that there was a positive significant association between AG genotype and T1DM (*P* = 0.01, *P* < 0.001) [22, 32]. One of these studies also revealed that there was a negative relation between AA genotype and T1DM [32].

Vitamin D receptor (VDR) was investigated in 2 studies [25, 26]. Both of them assessed the association of* Fok*I,* Bsm*I,* Apa*I, and* Taq*I variants of VDR with T1DM. In one study which was performed on 114 subjects, Aa (*P* = 0.003), FF (*P* = 0.008) and Bb (*P* = 0.014) genotypes were shown to be positively associated with T1DM but no significant association was observed between* Taq*I polymorphism and T1DM [25]. Another study which was performed on 187 subjects showed no significant association between T1DM and* Fok*I,* Bsm*I, and* Apa*I. However TT genotype was observed to be more frequent in controls (*P* = 0.007) and negatively associated with T1DM [26].

Different polymorphisms of TAP2 and their association with T1DM were investigated in 191 subjects. It was shown that Ile379Val and Stop687Gln had significant association with T1DM (*P* = 0.001 and *P* = 0.013, resp.). Other variants of TAP2 (transporter 2 ATP-binding cassette) including Ala565Thr, Arg651Cys, and Ala665Thr were not associated with T1DM significantly [27].

Association of alleles A2–A9 of CD4 with T1DM was assessed in 200 subjects. It was revealed that A3 allele had negative (*P* = 0.025) and A5 had positive (*P* = 0.001) significant association with T1DM [33].

Association of some other genes including osteopontin (rs1126772 polymorphism), integrin *α*4 (rs1449263 polymorphism), CD44 (rs8193 polymorphism), and TGF*β* (transforming growth factor) (+915C/G polymorphism) was also investigated in 2 studies and no significant relation was observed between these variants and T1DM [23, 29].

#### 3.2.2. MODY

In this category only one study was found [34] and the association of Val255Met polymorphism of HNF*α* and diabetes was investigated in 101 subjects including MODY subjects, their relatives, and healthy controls [34]. The study was designed as a case control association study and PCR-RFLP and direct sequencing was used for genotyping of variants. Val255Met mutation in this investigation was observed in MODY subjects and their relatives but not in control group.

#### 3.2.3. T2DM Related Genes

In this subgroup 42 studies were assessed and all of them were designed as case control association studies [22, 35–68, 70–75] except for one which was cross sectionally designed [69]. In 33 studies, PCR- RFLP was used for genotyping [22, 36–38, 40–54, 56, 58–61, 63–66, 69, 72–75]. In 5 of them PCR was used [35, 57, 62, 70, 71] and the remaining studies PCR-SCA [39], real-time PCR [55], ARMS-PCR [66], Gap PCR [67], and CSGE (conformation-sensitive gel electrophoresis) sequencing [68] as the genotyping method were applied.

Association between two polymorphisms of TCF7L2 including rs7903146 (C/T) and rs12255372 (G/T) was assessed in three studies [38, 48, 53]. In one study investigating rs7903146 (C/T) in 466 subjects, significant difference in the frequency of CC and CT genotypes was observed between T2DM and control group (*P* = 0.045) [38]. However in another study this polymorphism showed a positive significant association with TT genotype in T2DM (*P* = 0.008) [53]. Investigation of rs12255372 (G/T) polymorphism of TCF7L2 in another study showed that among 491 subjects there was a positive significant association between TT carriers and T2DM (*P* = 0.014) [48].

Investigating the association of calreticulin with T2DM in 650 subjects showed two mutations, only in case group, 9 bp deletion of 397–399 codons and G>T mutation at IVSII-142 [39].

Association of estrogen receptor *α* and its* Pvu*II(PP,Pp,pp) and* Xba*I(XX,Xx,xx) polymorphism with T2DM in two studies was assessed [40, 49]. In one study, the significant association of* Pvu*II and* Xba*I polymorphisms with T2DM was observed (*P* = 0.014 and *P* = 0.002, resp.) [40]. In another study it was revealed that pooled Pp+pp and XX+xx male carriers have positive significant association with T2DM (*P* = 0.001 and *P* = 0.026, resp.) [49].

Association of Pro12Ala polymorphism of PPAR*γ* with T2DM was investigated in three studies [41, 69, 75]. Negative significant association of Ala/Ala genotype with T2DM was observed in two studies (*P* = 0.003) [41, 75]. Ala/Pro carriers were shown to be positively associated in one study [41] and negatively associated in another study [75] with T2DM (*P* < 0.001 and *P* = 0.003, resp.). However in the third study no significant association was observed between Pro12Ala polymorphism of PPAR*γ* and T2DM [69].

Association of different polymorphisms of* ApoA*I with T2DM was investigated in two studies [43, 72]. In one study investigation of* MSP*I polymorphism showed no significant association with T2DM [43]. In another study it was shown that G-75A polymorphism of* ApoA*I was not associated with T2DM while a significant association was found between C+83T polymorphism and T2DM (*P* = 0.028) [72].

Two studies assessed the association of −156G>C polymorphism of CXCL5 with T2DM [44, 65]. In one study positive significant association of GC carriers (*P* = 0.006) and negative association of GG carriers (*P* = 0.01) was found between diabetic patients and healthy controls [44] and in another study positive significant association was found between GC or CC carriers and T2DM (*P* = 0.009) [64].

Adiponectin and its association with T2DM were investigated in two studies [56, 73]. In one study, two variants including +45T/G and −11391G/A were investigated but no significant relation was found between these two polymorphisms and T2DM [56]. However in another study investigating +45T/G polymorphism of adiponectin, it was revealed that nonobese T2DM subjects had significantly higher TT genotype (*P* = 0.04) [73].

In one study that investigated insulin receptor gene, the following mutations were found only in T2DM: 511C>A, 514T>G, 586, and 628T>A on exon 2, 694G>C, 680G>A on exon 3, 1627A>T on exon 8, AT>TG on intron 9, 2007C>C/T on exon 9, 2595C>C/T and 2669G>C/G on exon 13, 2706 and 2717C>G, 2752C>T, and 2753C>G on exon 14, and 3471T>A and 3516T>G on exon 19. These mutations were not seen in control subjects [68].

Other variants which had significant association with T2DM included haptoglobin (allele 2-2), leptin (G-2548A polymorphism), paraoxonase 2 (Ser311Cys polymorphism), GST (glutathione-S-transferase) M1 and GSTM1/GSTT1 interaction, calpain-10 (SNP (single nucleotide polymorphism) 43), IRS-1 (G972R polymorphism) and IRS-2 (G1057D polymorphism), VDR (vitamin D receptor) (TaqI polymorphism), KCNJ11 (E23K polymorphism), eNOS (endothelial nitric oxide synthase) VNTR (intron 4 a/b polymorphism), resistin (−420C/G polymorphism), and ACE (Insertion/Deletion) [35–37, 45, 47, 50, 55, 57, 58, 64, 70].

In contrast to above variants, CTLA4 (+49A/G polymorphism), HNF-1*α* (Ala98Val polymorphism), GLP-1R (glucagon-like peptid 1 receptor) (Thr149Met polymorohism), SLC30A8 (Arg325Trp polymorphism), GSTP1 (Ile105Val polymorphism), glucokinase (−30G/A polymorphism), SDF-1*β* (stromal derived factor -1 *β*) (G801A polymorphism), ApoE (apolipoprotein E), ENPP1 (ectoenzyme nucleotide pyrophosphate phosphodiesterase 1) (K121Q polymorphism), SUMO4 (small ubiquitin-like modifier 4) Met55Val polymorphism), MTHFR (C677T polymorphism), UCP2 (uncoupling protein 2) (−866G/A polymorphism), 5HTTLPR, IL-4 (−590C/T polymorphism), IFN-*γ* (interferon *γ*) (+874T/A polymorphism), CCR5 (C-C chemokine receptor type 5) (*δ*32mutation), HFE (hemochromatosis gene) (H63D, C282Y, PTPN1 (protein tyrosin Phosphatase 1B)/−51delA, −451A>G, −467T>C, −1023C>A, −1045G>A, −1286 3 bp del ACA, −1291 9 bp del CTAGACTAA polymorphisms) were not significantly associated with T2DM [22, 42, 46, 50–52, 54, 59–63, 66, 67, 71, 74].

#### 3.2.4. Genes Related to Insulin Resistance

In this subgroup, four studies were assessed and all were designed as case-control association studies. Three studies used PCR-RFL [76, 78, 79] and one study used real-time PCR [77] for genotyping.

The studies assessed in this subgroup showed a positive association of TG carriers of +45T/G polymorphism of adiponectin (*P* = 0.032) and Ala allele carriers of Pro12Ala polymorphism of PPAR*γ* (*P* = 0.036) with T2DM [76, 77]. A positive significant association of ff carriers of VDR was also shown (*Fok*I polymorphism) (*P* = 0.02) and negative association of 148insG carriers of PTPN1 (148insG polymorphism) (*P* = 0.041) with insulin resistance index [78, 79]. However no significant association was shown between adiponectin receptor-2/+795G/A and T2DM [76].

#### 3.2.5. Genes Related to Cardiovascular Disease in Diabetes

Design of all 11 studies in this subgroup was case-control association study [80–84, 86–90] except one study which was designed as cross sectional [85]. PCR-RFLP was used in all of them for genotyping [80–88, 90] except in one study which used ARMS-PCR method [89].

Paraoxonase 1 (163T/A polymorphism) was the only variant in this subgroup which was not significantly associated with atherosclerosis risk in T2DM [80]. Investigation of another polymorphism of Paraoxonase 1 (Q192R) revealed that diabetics with CAD had significant increase in RR genotype (*P* < 0.05) [81].

Investigating B1 and B2 alleles of CETP* Taq*I B allele in 400 subjects showed that pooling of B1B1+B1B2 and B1B1 genotypes had significant association with T2DM and CAD (*P* < 0.01). On the other hand assessing the G894T polymorphism of eNOS showed positive significant association between TG and TT carriers and CAD (*P* < 0.05). It was also shown that concomitant presence of NOS3 T allele and CEPT B1 allele was significantly associated with T2DM (*P* = 0.004) and CAD (*P* = 0.002) [83].

Investigation of apolipoprotein E (E2, E3, and E4) alleles showed the association of E2 and E4 alleles with CAD (*P* < 0.001) [84, 87]. Also it was shown that interaction of butyrylcholinesterase (BChE K) and apolipoprotein E (ApoE) was associated significantly with CAD and diabetes. ApoE4/BChE K had significant association with lipid profiles as well (*P* < 0.05) [87].

Adiponectin +276G/T and +45T/G were significantly associated with CAD (*P* = 0.023 and 0.033, resp.) [82] while investigating −1612 5A/6A polymorphism of MMP-3 (matrix metalloproteinase 3) showed a significant association with CAS (coronary artery stenosis) (*P* = 0.008) [86].

In addition A1166C polymorphism of AT1R (angiotensin I receptor) and −420C/G polymorphism of resistin in diabetic patients with CAD revealed significant association with diabetes, *P* = 0.01 and *P* = 0.009, respectively [84, 85].

Another study showed insertion(I)/deletion(D) polymorphism of angiotensin converting enzyme (ACE) DD genotype was significantly associated with higher blood pressure (*P* = 0.026) [89].

Factor V Leiden (G1691A polymorphism), prothrombin (G20210A polymorphism), and MTHFR (methylenetetrahydrofolate reductase) (C677T polymorphism) were the variants which were not significantly associated with CAD and T2DM in this subgroup [88].

#### 3.2.6. Genes Related to Diabetic Nephropathy

All of 15 studies in this subgroup were designed as case-control association studies [91–106]. In 7 studies, PCR-RFLP was used for genotyping [92–95, 99, 102, 103]; in four studies PCR was used [98, 101, 104, 105] and in 4 of them both PCR and PCR-RFLP were used [91, 96, 97, 100].

Studying the association of 163A/G polymorphism of SUMO4 showed a positive significant association between AA carriers and diabetic nephropathy (*P* < 0.05) [93].

Studying the interaction of eNOS/4a/b and G894T revealed positive significant association between 4a or 894T allele carriers and macro- and microalbuminuria (*P* = 0.01, *P* = 0.02, resp.) [91]. It was observed that the interaction of eNOS (G894T polymorphism) and ACE (I/D alleles) was significantly associated with more frequent macroalbuminuria (*P* = 0.035) [97].

It has also been shown that interaction of eNOS (G894T polymorphisms) and MTHFR (C677T and A1298C polymorphisms), eNOS T/1298 C, and eNOS T/677 T carriers had positive significant association with macroalbuminuria. In addition significant association of eNOS GT+TT carriers with lipid profile (*P* < 0.05) was shown in this study [99].

C677T and A1298C polymorphisms of MTHFR were also investigated in another study revealing that 677T, 1298C, and 677T/1298C carriers were significantly associated with macroalbuminuria (*P* < 0.001). Positive significant association when pooling CT+TT genotypes and 677T/1298C was also found with microalbuminuria (*P* < 0.001) [103]. Investigating the interaction of MTHFR (A1298C, C677T polymorphisms) and ACE (I/D alleles) showed a positive significant association of ACE D/677T (*P* = 0.035) and ACE D/1298C (*P* = 0.012) genotypes with macroalbuminuria [96].

ACE (I/D alleles) polymorphisms were investigated in three studies. In one study positive significant association (*P* < 0.01) between DD carriers and progression but not development of albuminuria was observed [104]. In another study in nephropathic subjects, association of DD carriers and T2DM (*P* = 0.006) but not nephropathy was revealed [105]. However another study did not show significant association of ACE with macroalbuminuria [98]. Interaction of ACE (I/D alleles) with Factor V Leiden (G1691A polymorphism) was not significant [100].

Association of interleukins including IL-4 and IL-10 with diabetic nephropathy was also investigated in two studies. Study of −592C/A polymorphism of IL-10 revealed positive significant association between CC carriers and nephropathy (*P* = 0.001) [94] and study of −590C/T polymorphism of IL-4 revealed positive significant association between CT carriers and nephropathy (*P* < 0.001) [101].

Other significant associations between variants and diabetic nephropathy included AT2R (angiotensin II receptor) (−1332G/A polymorphism), VEGF (vascular epithelium growth factor) (+405G/C polymorphism), and VDR (*Taq*I polymorphism) [92, 95, 102].* Apa*I polymorphism of VDR was not significantly associated with diabetic nephropathy [102].

#### 3.2.7. Genes Related to Diabetic Retinopathy

All of the 3 studies in this subgroup were conducted as case-control association studies [106–108].

Investigating null/positive genotype of GSTM1 in a study, which used ARMS-PCR for genotyping, revealed that null genotype is significantly associated with diabetic retinopathy (*P* < 0.05) [106]. This result was confirmed in another study by using multiplex PCR as genotyping [107], although no significant association between GSTT1 and retinopathy was found in this study [107].

Investigating +405G/C polymorphism of VEGF also revealed positive significant association between GG carriers and diabetic retinopathy (*P* = 0.005) [108].

#### 3.2.8. Genes Related to Diabetic Foot Ulcer

In this subgroup only one study was found [109] which was case-control designed and used ARMS-PCR for genotyping. In this study association of −7C/T and −2578C/A polymorphisms of VEGF with diabetic foot ulcer (DFU) was investigated, and a positive significant association between AA carriers and DFU (*P* = 0.03) was observed. But no significant association was found between −7C/T of VEGF and DFU [109].

## 4. Discussion

Diabetes mellitus is the eighth most frequent disease leading cause of death throughout the world and now ranks the fifth, following communicable diseases, cardiovascular disease, cancer, and injuries [110]. Prevalence of diabetes mellitus is increasing worldwide [6].

Type 2 diabetes is the most frequent type of diabetes mellitus [110, 111]. Multiple genes and environmental factors affect the prevalence of T2DM. Systematic review and meta-analysis studies have assessed the association between different genes and T2DM. Glutathione-S-transferase including GSTMI, GSTM1, and GSTP1 are important genes and their association with diabetes has been investigated in two meta-analysis studies [112, 113]. It was revealed that null genotype of both GSTM1 and GSTT1 could be as a risk factor for diabetes while no significant association was found between GSTP1 and diabetes [112, 113]. These results are in line with our finding and confirming the association of the null genotype of GSTT1 and GSTM1 with T2DM in Iranian population [50]. The relation between GSTT1 and GSTM1 and diabetic retinopathy has also been shown in Iranian population [106, 107].

MTHFR is another important gene in association with T2DM and other related complications. No association of C677T polymorphism of MTHFR gene was found in Africans, Asians, and Caucasians [114]. MTHFR is a gene which is involved in DNA methylation and synthesis and regulated folate activity. It has been reported that mutant homozygote and heterozygote of C677T polymorphism of MTHFR increase plasma homocysteine which is an important factor leading to diabetic nephropathy (DN) and, as a result, C677T polymorphism of MTHFR can be associated with the development of DN. In Caucasians this association has also been observed [115]. In Iranian population, in line with other populations, no significant association was found between MTHFR polymorphisms and T2DM [63] although significant association of 677T and 1298C alleles with diabetic nephropathy was observed [87]. Interaction of two polymorphisms and also interaction of MTHFR polymorphisms with other genes such as ACE and eNOS showed an association with development of DN [96, 99].

Interleukins especially IL-10 are shown to have significant association with T2DM in different ethnic groups. Significant association between −592C/A and −819C/T polymorphisms of IL-10 and T2DM risk in Africans was shown [116]. Also −1082A/G polymorphism of IL-10 seems to be a risk factor for T2DM in Asians but not in Europeans and Africans [116]. In another study it was reported that −1082GG genotype of IL-10 and −174CC genotype of IL-6 are as risk factors for T2DM in Egyptians [117]. In our study IL-4 has been studied but no significant association with T2DM was seen [66].

Investigation of different polymorphisms of adiponectin and their association with T2DM in a meta-analysis study revealed −11391G>A and −11426A>G polymorphisms of adiponectin as risk factor for T2DM in Europeans and −11377C>G variant of adiponectin as risk factor of T2DM in Europeans and Asians [110]. It was also shown that +45T>G and +276G>T variants are not associated with T2DM in Europeans, Africans, and Asians [110]. In contrast to previous studies, +45T>G and −11391G>A polymorphisms were not associated with T2DM in this study due to difference in genetic background [56].

Among candidate genes in association with T2DM, TCF7L2 is one of the strongest genes related to diabetes. In a meta-analysis study after pooling all data of European, African, and Asian populations, it has been revealed that rs12255372 polymorphism of TCF7L2 significantly increases the risk of T2DM. However no data from Iranian population as a type of Asian population was included in this meta-analysis [111]. In line with these data, in our study, we found a positive association between rs12255372 and rs7903146 variants of TCF7L2 and T2DM [38, 48, 53].

Pro12Ala polymorphism of PPAR*γ* is found to be as a protective variant especially in Asian population although the results were highly heterogeneous [118]. In our study the results were controversial. In one study no association was found between this polymorphism and T2DM in contrast with previous studies [69] while in other studies protective effect of this polymorphism with T2DM was confirmed in line with previous studies [41, 75]. These controversial data may be due to different ethnicity in different populations being studied in Iran. Small sample size could be another reason for the controversial results.

Type 1 diabetes mellitus is another type of diabetes in which HLA (human lymphocyte antigen), IR1R1 (interleukin-1 receptor type 1), CTLA-4, and VDR are some important genes studied in association with T1DM [119]. VDR is one of the most studied genes in relation to T1DM due to its role in T-cell mediated autoimmune diseases [119, 120]. Meta-analysis studies showed that* Bsm*I and* Fok*I polymorphisms of VDR increased the risk of T1DM in East and West Asians, respectively [119]. In our study, controversial results were found. In one study* Bsm*I was shown to be associated with T1DM [25] while in another study no association was found [26].* Fok*I was not associated with T1DM in our study in contrast to previous studies which might be due to difference in ethnicity and genetic background or insufficient clinical data [25, 26].

Among diabetic complications, CAD and DN are the most studied complications. Meta-analysis studies showed that rs2010963 and rs3025039 polymorphisms of VEGF, 4b/a, T-786C, and G894T polymorphisms of eNOS and ACE are associated with DN [121–123]. In our study, 4a/b and G894T polymorphisms of eNOS and ACE were shown to be associated with DN in line with previous studies [91, 104, 105]. In our study rs2010963 polymorphism of VEGF was also found to be associated with diabetic nephropathy which was in line with previous studies [95].

The most investigated genes in association with CAD include PPAR*γ*, TCF7L2, ACE, TNF-*α*, adiponectin, and IRS1 [118]. In our study, +45T>G and +276G>T polymorphisms of adiponectin were investigated and the significant association of these variants with CAD was confirmed [82].

Overall it seems that more studies are needed to identify diabetes susceptibility genes in Iranian population. Investigation of candidate genes is one way to understand these genes, but the method with the least expenses and error is GWAS which scans a set of loci in association with a disease in many people. GWAS in different populations such as Americans, Caucasians, Australians, West Africans, and Europeans revealed multiple loci in association with T2DM [124–128]. In Asians especially in Middle Eastern countries GWAS is critically needed. In our investigations, we reviewed five studies which investigated the association between eNOS, ACE, MTHFR, and factor V Leiden and the interaction between them and diabetic nephropathy and reach a coherent result [91, 97, 99, 100, 104] while GWAS could be helpful and reduce time and other expenses [126].

However, there are some strength and some limitations in our study. Firstly, this systematic review, for the first time, assessed the association between genetic variants and diabetes in Iranian population. Moreover, the studies included in this systematic review were assessed for quality and selected as high quality (scored ≥ 3). For limitations it should be considered that all included studies were observational, which does not allow reliable inferences about causality. Moreover due to methodological heterogeneity, we were not able to pool the data and perform the meta-analysis.

## 5. Conclusion

Our study showed significant association between CTLA-4, IL-18, VDR, TAP2, IL-12, and CD4 genes and T1DM. HNF*α* gene had significant association with MODY. The following genes showed significant association with T2DM: haptoglobin, paraoxonase, leptin, TCF7L2, calreticulin, ER*α*, PPAR-*γ*2, CXCL5, calpain-10, IRS-1 and 2, GSTM1, KCNJ11, eNOS, VDR, resistin, INSR, ACE, ApoA-I, adiponectin, and PTPN1. In assessment of association between CAD and diabetes in Iranian population the following genes showed significant association: paraoxonase 1, adiponectin, eNOS, CETP, AT1R, resistin, MMP-3, BChE K, Apo E, ACE. The following genes had significant association with diabetic nephropathy: eNOS, AT2R, SUMO4, IL-10, VEGF, MTHFR, ACE, and VDR. The following genes had significant association with diabetic retinopathy: GSTM1 and VEGF and also VEGF had significant association with diabetic foot ulcer.

Insufficient data might cause the conflicting results; therefore GWAS on defined population with large sample size is suggested as a more comprehensive approach answering many more questions.

## Figures and Tables

**Table 1:** Characteristics of included articles in systematic review.

Reference	Genes/*SNP or allele *	Patients' characteristics	City	Study design	Method	Significant association
T1DM and complications						
Ahmadi et al., 2013 [22]	CTLA-4/*+49A/G *	60 T1DM, 56 T2DM, and 107 healthy, male (M)/female (F)	Kurdistan	Case control	PCR-RFLP	Positive significant association between AG carriers and T1DM (*P* = 0.01)
Karamizadeh et al., 2013 [23]	(i) Osteopontin/*rs1126772* (ii) Integrin *α*4/*rs1449263* CD44/*rs8193 *	87 T1DM and 86 healthy, <20 yr, M/F	Shiraz	Case control	PCR-RFLP	Nonsignificance
Rahbani-Nabar et al., 2013 [24]	IL-18/*−137C/G *	104 T1DM and 92 healthy, 9–32 yr, M/F	Tabriz	Case control	SS-PCR	Positive significant association between GG carriers and T1DM (*P* = 0.037)
Bonakdaran et al., 2012 [25]	(i) VDR/*FokI (FF, Ff, ff),* (ii) *BsmI (BB, Bb, bb), ApaI (AA, Aa, aa), * (iii) *TaqI (TT, Tt, tt) *	69 T1DM and 45 healthy, <35 yr, M/F	Mashhad	Case control	PCR-RFLP	Positive significant association between Aa (*P* = 0.003), FF (*P* = 0.008), and Bb (*P* = 0.014) carriers with T1DM, significant association between ff genotype and history of ketoacidosis (*P* = 0.04)
Mohammadnejad et al., 2012 [26]	(i) VDR/*TaqI (TT, Tt, tt)* (ii) VDR, *FokI, BsmI, ApaI *	87 T1DM and 100 healthy, 17–38 yr, M/F	Mashhad	Case control	PCR-RFLP	(i) Negative significant association between TT carriers and T1DM (*P* = 0.007) (ii) Nonsignificance association between other carriers of VDR and T1DM
Afshari et al., 2011 [27]	(i) TAP2/*Ile379Val* (ii) TAP2/*Stop687Gln* (iii) TAP2/*Ala565Thr, Arg651Cys, Ala665Thr *	87 T1DM and 104 healthy, <30 yr, M/F	Mashhad	Case control	ARMS-PCR	(i) Significant association between Ile379Val and T1DM (*P* = 0.001) (ii) Significant association between Stop687Gln and T1DM (*P* = 0.013) (iii) Nonsignificant association between other carriers of TAP2 and T1DM
Massoud et al., 2009 [28]	(i) IL-18/*−137C/G* (ii) IL-18/*−607A/C *	75 T1DM and 88 healthy, <30 yr, M/F	Tehran	Case control	PCR-SSP	(i) Positive significant association between GG carriers and T1DM (*P* = 0.0001) (ii) Significant association between CC genotype with T1DM (*P* = 0.0001) (iii) Nonsignificance association between other carriers of IL-18 and T1DM
Masoud et al., 2007 [29]	TGF*β*/*+915C/G *	75 T1DM and 88 healthy, <30 yr, M/F	Tehran	Case control	PCR-SSP	Nonsignificance
Masoud et al., 2007 [30]	IL-12/*+1188A/C *	75 T1DM and 88 healthy, <30 yr, M/F	Tehran	Case control	PCR-SSP	Significant association between AA and AC genotype and T1DM (*P* = 0.035)
Mojtahedi et al., 2006 [31]	IL-18/*−607A/C, * *−137C/G *	112 T1DM and 194 non-DM, <15 and >15 yr, M/F	Shiraz	Case control	PCR-SSP	−137CC and −607AA/−137CC significant association with T1DM in onset >15 yr (*P* = 0.027)
Mojtahedi et al., 2005 [32]	CTLA-4/*+49A/G *	109 T1DM and 331 healthy, 0–37 yr, M/F	Shiraz	Case control	PCR-SSP PCR-RFLP	(i) Positive significant association between AG carriers and T1DM (*P* = 0.00) (ii) Negative significant association between AA carriers and T1DM (*P* = 0.00)
Zamani et al., 2005 [33]	CD4/*A2–A9 *	92 T1DM and 108 healthy, >35 yr, M/F	Tehran	Case control	PCR	Negative significant association between A3 allele and T1DM (*P* = 0.025) Positive significant association between A5 allele and T1DM (*P* = 0.001)

MODY						
Taghavi et al., 2009 [34]	HNF*α*/*Val255Met *	30 MODY, 21 relatives, and 50 healthy, 25–35 yr, M/F	Mashhad	Case control	PCR-FLP sequencing	The mutation was found in patients and relatives but not in controls

T2DM and complications						
Ahmadi et al., 2013 [22]	CTLA-4/*+49A/G *	Said above	Said above	Said above	Said above	Nonsignificance
Amiri et al., 2013 [35]	Haptoglobin/*1-1, 2-1, 2-2 *	134 T2DM with MVCs, 71 T2DM without MVCs, 46–72 yr, M/F	Sari	Case control	PCR	Hp2-2 highly significant for T2DM (*P* = 0.05)
Andalib et al., 2013 [36]	Paraoxonase 2/*Ser311Cys *	100 T2DM and 100 healthy, 51 yr, M/F	Isfahan	Case control	PCR-RFLP	Positive significant association of Cys/Cys and Cys/Ser carriers and negative significant association of Ser/Ser carriers with T2DM (*P* < 0.05)
Kohan et al., 2013 [37]	Leptin/*G-2548A *	100 T2DM and 100 healthy, 44–66 yr, M/F	Arsanjan	Case control	PCR-RFLP	Positive significant association between GG carriers and T2DM (*P* = 0.004)
Alami et al., 2013 [38]	TCF7L2/*rs7903146 (C/T) *	233T2DM and 233 controls, >40 yr, M/F	Gorgan	Case control	PCR-RFLP	CC and CT genotypes significant difference between T2DM and controls (*P* = 0.045)
Mahmazi et al., 2013 [39]	Calreticulin	120 T2DM and 530 controls, 43–66 yr, M/F	Zanjan	Case control	PCR-SCA	9 bp deletion of 397–399 codons G>T mutation at IVSII-142 in T2DM not in controls
Mohammadi et al., 2013 [40]	(i) ER*α*/*PvuII (PP, Pp, pp)* (ii) ER*α*/*XbaI (XX, Xx, xx) *	174 T2DM and 174 ND, 35–65 yr, M/F	Jahrom	Case control	PCR-RFLP	(i) Significant association with T2DM (*P* = 0.014), TC (*P* = 0.007), and TG (*P* = 0.005) female carriers (ii) Significant association with T2DM (*P* = 0.002), TC (*P* = 0.003), and TG (*P* = 0.009) female carriers
Motavallian et al., 2013 [41]	PPAR-*γ*2/*Pro12Ala *	100 T2DM and 100 healthy, 51 yr, M/F	Isfahan	Case control	PCR-RFLP	Negative significant association between Ala/Ala carriers (*P* < 0.001) and positive significant association between Ala/Pro carriers with T2DM (*P* < 0.001)
Sepahi et al., 2013 [42]	HNF-1*α*/*Ala98Val* GLP-1R/*Thr149Met *	100 T2DM and 50 healthy controls, ≤35 and >35 yr, M/F	Mashhad	Case control	PCR-RFLP	Nonsignificance
Sheikhha et al., 2013 [43]	APOA1/*MSP-I *	200 T2DM and 200 healthy, 41.8 yr, M/F	Yazd	Case control	PCR-RFLP	Nonsignificance
Yaghoubi et al., 2013 [44]	CXCL5/*−156G > C *	100 T2DM, 54 yr and 100 healthy, 56 yr, M/F	Ardabil	Case control	PCR-RFLP	Positive significant association of GC carriers (*P* = 0.006) and negative significant association of GG carriers with T2DM (*P* = 0.01)
Bahreini et al., 2012 [45]	Calpain-10/*SNP43(A/G) *	102 T2DM and 100 healthy, 40–70 yr, M	East Azerbayjan	Case control	PCR-RFLP	G allele as risk factor of T2DM (*P* = 0.037)
Derakhshan et al., 2012 [46]	SDF-1*β*/*G801A *	200 T2DM and 200 healthy, 40 yr, M/F	Rafsanjan	Case control	PCR-RFLP	Nonsignificance
Haghani et al., 2012 [47]	(i) IRS-1/*G972R* (ii) IRS-2/*G1057D *	336 T2DM and 341 healthy, 44–63 yr, M/F	Ilam and Kermanshah	Case control	PCR-RFLP	(i) Positive significant association of GR (*P* = 0.001) and RR (*P* = 0.0001) carriers with T2DM (ii) Positive significant association between GD carriers and T2DM (*P* = 0.016)
Alami et al., 2012 [48]	TCF7L2/*rs12255372 (G/T) *	236 T2DM and 255 healthy, >37 yr, M/F	Gorgan	Case control	PCR-RFLP	Positive significant association between TT carriers and T2DM (*P* = 0.014)
Meshkani et al., 2012 [49]	(i) ER*α*/*PvuII (PP, Pp, pp)* (ii) ER*α*/*XbaI (XX, Xx, xx) *	155 T2DM and 377 controls, 23–79 yr, M/F	Tehran	Case control	PCR-RFLP	(i) Positive significant association of pooled Pp + pp male carriers (*P* = 0.001) with T2DM (ii) Positive significant association of pooled XX+xx male carriers (*P* = 0.026) with T2DM
Moasser et al., 2012 [50]	GSTM1*/present/null* GSTT1*/present/null* GSTP1*/Ile105Val *	171 T2DM and 169 healthy, 25–65 yr, M/F	Shiraz	Case control	PCR-RFLP	GSTM1-null (*P* = 0.016) and interaction of GSTM-null/GSTT1-null (*P* = 0.022) significant association with T2DM
Mohaddes et al., 2012 [51]	SLC30A8/*Arg325Trp *	125 T2DM and 125 controls, 40–70 yr, M/F	Azarbayjan	Case control	PCR-RFLP	Nonsignificance
Oladi et al., 2012 [52]	Glucokinase/*−30G/A *	542 subjects, 18–65 yr, M/F	Mashhad	Cross sectional	PCR-RFLP	Nonsignificance
Palizban et al., 2012 [53]	TCF7L2/*rs7903146 (C/T) *	110 T2DM and 80 healthy, 46–67 yr, M/F	Isfahan	Case control	PCR-RFLP	Positive significant association between TT carriers and T2DM (*P* = 0.008)
Tabatabaei-Malazy et al., 2012 [54]	ApoE/*E3-E*3, E2-E3, *E4-E3 *	156 T2DM and 155 healthy, 25–65, M/F	Tehran	Case control	PCR-RFLP	Nonsignificance
Ghasemi et al., 2012 [55]	KCNJ11/*E23K *	358 T2DM and 388 healthy, 41–69 yr, M/F	Rasht	Case control	Real time PCR	Positive significant association between KK carriers and obese T2DM (*P* = 0.037)
Ranjbar et al., 2011 [56]	Adiponectin/*+45T/G,* *−11391G/A *	244 T2DM and 99 healthy, 37–65 yr, M/F	Rafsanjan	Case control	PCR-RFLP	Nonsignificant
Mehrab-Mohseni et al., 2011 [57]	eNOS VNTR/*intron 4 a/b *	220 T2DM and 96 healthy, 53 ± 15 yr, M/F	Rafsanjan	Case control	PCR	Positive significant association between aa or ab carriers and T2DM (*P* = 0.02)
Nosratabadi et al., 2011 [58]	(i) VDR/*TaqI (TT/Tt/tt)* (ii) VDR/*ApaI (AA/Aa/aa) *	100 T2DM and 100 healthy, 40 yr, M/F	Rafsanjan	Case control	PCR-RFLP	(i) Positive significant association between Tt carriers and T2DM (*P* < 0.001) (ii) Nonsignificant association between ApaI carriers and T2DM
Saberi et al., 2011 [59]	ENPP1/*K121Q *	155 T2DM and 377 healthy, 23–79 yr, M/F	Tehran	Case control	PCR-RFLP	Nonsignificance
Fallah et al., 2010 [60]	SUMO4/*Met55Val (163A/G) *	50 T2DM and 50 healthy, 25–45 yr, M/F	Tehran	Case control	PCR-RFLP	Nonsignificance
Heidari et al., 2010 [61]	UCP2/*−866G/A *	75 T2DM, 75 ND obese and 75 ND nonobese, 35–76 yr, M/F	Tehran	Case control	PCR-RFLP	Nonsignificance
Nazem et al., 2010 [62]	5HTTLPR/*SS, SL, LL *	90 T2DM and 90 healthy, 54–66 yr, M/F	Shiraz	Case control	PCR	Nonsignificance
Bazzaz et al., 2010 [63]	MTHFR/*C677T *	401 T2DM, 74 ND obese and 207 ND nonobese, 30–63 yr, M/F	Tehran	Case control	PCR-RFLP	Nonsignificance
Emamgholipour et al., 2009 [64]	resistin/*−420C/G *	47 T2DM and 66 healthy, 58 ± 9 yr, M/F	Tehran	Case control	PCR-RFLP	Positive significant association between CC carriers and T2DM (*P* = 0.009)
Hasani-Ranjbar et al., 2009 [65]	CXCL5/*−156G/C *	230 T2DM and 120 healthy, 40–63 yr, M/F	Rafsanjan	Case control	PCR-RFLP	Positive significant association between GC or CC carriers and T2DM (*P* = 0.004)
Kazemi Arababadi et al., 2009 [66]	IL-4/*−590C/T* IFN-*γ*/*+874T/A *	160 T2DM and 160 healthy, 38 ± 9 yr, M/F	Rafsanjan	Case control	PCR-RFLP ARMS-PCR	Nonsignificance
Arababadi et al., 2009 [67]	CCR5/*δ32mutation *	200 T2DM and 300 healthy, 40 ± 9 yr, M/F	Rafsanjan	Case control	Gap-PCR	Nonsignificance
Kazemi et al., 2009 [68]	INSR	128 T2DM, >40 yr, M/F	Tehran	Case control	PCR CSGE sequencing	Following mutations were found only in T2DM 511C>A, 514T>G, 586, and 628T>A on exon 2 694G>C and 680G>A on exon 3 1627A>T on exon 8 AT> TG on intron 9 2007C>C/T on exon 9 2595C>C/T and 2669G>C/G on exon 13 2706 and 2717C>G, 2752C>T, 2753C>G, on exon 14 3471T>A and 3516T>G on exon 19
Mirzaei et al., 2009 [69]	PPAR*γ*2/*Pro12Ala *	78 normal, 78 obese, 78 T2DM, and 78 obese T2DM, 25–64 yr, M/F	Tehran	Cross sectional	PCR-RFLP	Nonsignificance
Nikzamir et al., 2008 [70]	ACE/*insertion (I)/deletion (D) *	170 T2DM and 144 healthy, M/F	Tehran	Case control	PCR	Positive significant association between DD carriers and T2DM (*P* = 0.02)
Sharifi et al., 2008 [71]	HFE/*H63D, C282Y *	101 T2DM and 101 healthy, 55 ± 11 yr, M/F	Zanjan	Case control	PCR	Nonsignificance
Besharati et al., 2007 [72]	(i) ApoA-I/*G-75A* (ii) ApoA-I/*C+83T *	215 subjects, 26–64 yr, M/F	Tehran	Cross sectional	PCR-RFLP	(i) Nonsignificant association between G-75A carriers and T2DM (ii) Positive significant association between CT carriers and T2DM (*P* = 0.028)
Hasani-Ranjbar et al., 2007 [73]	Adiponectin/*+45T/G *	80 T2DM obese, 72 T2DM nonobese, and 70 healthy, 25–64 yr, M/F	Tehran	Case control	PCR-RFLP	Positive significant association between TT carriers and nonobese T2DM (*P* = 0.04)
Meshkani et al., 2007 [74]	PTPN1/*−51delA, * *−451A>G, −467T>C* *−1023C>A,* *−1045G>A, −1286 3 bp del ACA, −1291 9 bp del CTAGACTAA *	174 T2DM and 412 healthy, 23–79, M/F	Tehran	Case control	PCR sequencing PCR-RFLP	Nonsignificance
Meshkani et al., 2007 [75]	PPAR*γ*/*Pro12Ala *	412 T2DM and 284 healthy, 23–79 yr, M/F	Tehran	Case control	PCR-RFLP	Negative significant association between Pro/Ala or Ala/Ala carriers and T2DM (*P* = 0.003)

T2DM patients and insulin resistance						
Namvaran et al., 2012 [76]	(i) Adiponectin/*+45T/G* (ii) Adiponectin receptor-2/*+795G/A *	101 T2DM and 128 healthy, 30–70 yr, M/F	Shiraz	Case control	PCR-RFLP	(i) Positive significant association between TG carriers and T2DM (*P* = 0.032) (ii) Nonsignificant association between +794G/A carriers and insulin resistance
Namvaran et al., 2011 [77]	PPAR*γ*/*Pro12Ala *	101 T2DM and 128 healthy, 30–70 yr, M/F	Shiraz	Case control	Real time PCR	Positive significant association between Ala allele carriers and T2DM (*P* = 0.036)
Hossein-nezhad et al., 2009 [78]	VDR/*FokI (FF, Ff, ff) *	105 T2DM, 55 ± 10 yr, M/F	Tehran	Case series	PCR-RFLP	Positive significant association between ff carriers and insulin resistance index (*P* = 0.02)
Moosapoor et al., 2007 [79]	PTPN1/*148insG *	71 T2DM and 264 ND, 20–80 yr, M/F	Tehran	Case control	PCR-RFLP	Negative significant association between 148insG carriers and insulin resistance index (*P* = 0.041)

T2DM patients and heart diseases						
Bayatmakoo et al., 2013 [80]	Paraoxonase 1/*163T/A* *(L55M) *	105 CAD/DM and 95 CAD/ND, <85 yr, M/F	Tabriz	Case control	PCR-RFLP	Nonsignificance
Bayatmakoo et al., 2012 [81]	Paraoxonase 1/*575G>A* *(Q192R) *	105 DM/CAD and 95 CAD/ND, <85 yr, M/F	Tabriz	Case control	PCR-RFLP	Positive significant association between RR carriers and CAD/DM (*P* < 0.05)
Esteghamati et al., 2012 [82]	(i) Adiponectin/*+45T/G* (ii) Adiponectin*/+276G/T *	114 CAD/DM and 127 DM, 42–71 yr, M/F	Tehran	Case control	PCR-RFLP	(i) Negative significant association between 45TT carriers and CAD (*P* = 0.033) (ii) Positive significant association between 276GG carriers and CAD (*P* = 0.023)
Rahimi et al., 2012 [83]	eNOS/*G894T* CETP/*B1 *	102 CAD/DM, 105 CAD/ND, 101 DM, and 92 ND, 45–66 yr, M/F	Kermanshah	Case control	PCR-RFLP	Positive significant association of concomitant presence of NOS3 T allele and CEPT B1 allele with T2DM (*P* = 0.004) and CAD (*P* = 0.002)
Assali et al., 2011 [84]	AT1R/*A1166C *	145 CAD/DM and 164 CAD, <50 and ≥50 yr, M/F	Mashhad	Case control	PCR-RFLP	Positive significant association of AC and CC carriers with DM (*P* = 0.01)
Emamgholipour et al., 2009 [85]	Resistin/*−420C/G *	113 CAD with and without DM, 58 ± 9 yr, M/F	Tehran	Cross sectional	PCR-RFLP	Positive significant association between CC carriers and DM (*P* = 0.009)
Fallah et al., 2010 [86]	MMP-3/*−1612 5A/6A *	305 CAD/DM and 313 DM, 61 ± 9 yr, M/F	Tehran	Case control	PCR-RFLP	Positive significant association between 6A/6A carriers and CAS (*P* = 0.008)
Vaisi-Raygani et al., 2010 [87]	BChE K/*G1615A* APO E/*E2, E3, E4 *	118 DM, 162 CAD/ND, 172 DM/CAD, and 179 healthy, 42–68 yr, M/F	Kermanshah	Case control	PCR-RFLP	Positive significant association of GA, AA, and E4 carriers with CAD and DM (*P* < 0.05) Positive significant association of BChE K/ApoE4 carriers with CAD and DM (*P* < 0.05) Significant association of BChE K/ApoE4 with lipid profile (*P* < 0.05)
Rahimi et al., 2009 [88]	Factor V Leiden/*G1691A* Prothrombin/*G20210A* MTHFR/*C677T *	65 CAD/DM, 52 CAD/ND, and 59 healthy, 46064 yr, M/F	Kermanshah	Case control	PCR-RFLP	Nonsignificance
Nakhjavani et al., 2007 [89]	ACE/*I/D *	82 DM with hypertension and 87 DM without hypertension, 49–63 yr, M/F	Tehran	Case control	PCR	Positive significant association between DD carriers and hypertension (*P* = 0.026)
Vaisi-Raygani et al., 2007 [90]	Apolipoprotein/*E2, E3, E4 *	152 CAD/DM, 262 CAD/ND, and 300 healthy, 35–73 yr, M/F	Kermanshah	Case control	PCR-RFLP	Positive significant association of E2 and E4 allele carriers with CAD (*P* < 0.001)

T2DM patients and nephropathy						
Rahimi et al., 2013 [91]	eNOS/*4a/b, G894T *	63T2DM/microalbuminuria, 57T2DM/macroalbuminuria, 52T2DM/normoalbuminuria, 121 DN, and 101 healthy, 45–66 yr, M/F	Kermanshah	Case control	PCR PCR-RFLP	Positive significant association of 4a or 894T allele carriers and macro- (*P* = 0.01) or microalbuminuria (*P* = 0.02)
Rahimi et al., 2013 [92]	AT2R/*−1332G/A *	28T2DM/microalbuminuria, 22T2DM/macroalbuminuria, 20T2DM/normoalbuminuria, and 112 healthy, 43–63 yr, M/F	Kermanshah	Case control	PCR-RFLP	Positive significant association between AA carriers and nephropathy (*P* = 0.016)
Shahsavar et al., 2013 [93]	SUMO4/*163A/G* *(M55V) *	50 T2D/DN and 50 T2DM non-DN, 25–45 yr, M/F	Tehran	Case control	PCR-RFLP	Positive significant association between AA carriers and nephropathy (*P* < 0.05)
Arababadi et al., 2012 [94]	IL-10/*−592C/A *	100 T2DM/non-DN, 100 T2DM/DN and 100 healthy, 31–49 yr, M/F	Rafsanjan	Case control	PCR-RFLP	Positive significant association between CC carriers and DN (*P* = 0.001)
Nikzamir et al., 2012 [95]	VEGF/*+405G/C *	255 T2DM/microalbuminuria and 235 T2DM/nonalbuminuric, 50–67 yr, M/F	Tehran	Case control	PCR-RFLP	Positive significant association between GG carriers and albuminuria (*P* = 0.002)
Rahimi et al., 2012 [96]	MTHFR/*A1298C, C677T* ACE/*I/D *	72T2DM/MicAlb, 68T2DM/MacAlband 72 T2DM/non-DN, 46–65 yr, M/F	Kermanshah	Case control	PCR-RFLP PCR	Positive significant association of ACE D/677T (*P* = 0.035) and ACE D/1298C (*P* = 0.012) carriers with macroalbuminuria
Rahimi et al., 2012 [97]	eNOS/*G894T* ACE/*I/D *	72T2DM/microalbuminuria, 68T2DM/macroalbuminuria and 72 T2DM/non-DN, 46–65 yr, M/F	Kermanshah	Case control	PCR-RFLP PCR	Positive significant association between ACE D carriers and macroalbuminuria (*P* = 0.035)
Felehgari et al., 2011 [98]	ACE/*I/D *	68 T2DM/macroalbuminuria and 72 T2Dm/normoalbuminuria, 46–65 yr, M/F	Kermanshah	Case control	PCR	Nonsignificance
Jafari et al., 2011 [99]	eNOS/*G894T* MTHFR/*C677T, A1298C *	72T2DM/microalbuminuria, 68T2DM/macroalbuminuria and 72 T2DM/non-DN, 46–65 yr, M/F	Kermanshah	Case control	PCR-RFLP	(i) Positive significant association of eNOS T/1298 C and eNOS T/677 T carriers with macroalbuminuria (*P* < 0.05) (ii) Significant association of eNOS GT+TT carriers with lipid profile (*P* < 0.05)
Rahimi et al., 2011 [100]	ACE/*I/D* Factor V Leiden/*G1691A *	217/mean 55/both	Kermanshah	Case control	PCR PCR-RFLP	Nonsignificance
Arababadi, 2010 [101]	IL-4/*−590C/T *	100 T2DM/DN and 150 healthy, 33–47 yr, M/F	Rafsanjan	Case control	PCR	Positive significant association between CT carriers and DN (*P* < 0.001)
Nosratabadi et al., 2010 [102]	(i) VDR/*TaqI (TT/Tt/tt)* (ii) VDR/*ApaI (AA/Aa/aa) *	100 T2DM/non-DN, 100 T2DM/DN, and 100 healthy, 31–49 yr, M/F	Rafsanjan	Case control	PCR-RFLP	(i) Positive significant association between Tt carriers and DN (*P* = 0.012) (ii) Nonsignificant association between ApaI carriers and DN
Rahimi et al., 2010 [103]	MTHFR/*C677T, A1298C *	72T2DM/microlbumiunira, 68T2DM/macroalbuminuria, and 72 T2DM/non-DN, 46–65 yr, M/F	Kermanshah	Case control	PCR-RFLP	(i) Positive significant association of 677T, 1298C and 677T/1298C carriers with macroalbuminoria (*P* < 0.001) (ii) Positive significant association of CT+TT and 677T/1298C carriers with microalbuminuria (*P* < 0.001)
Nikzamir et al., 2009 [104]	ACE/*I/D *	129T2DM/microlbumiunira, 48T2DM/macroalbuminuria, and 145T2DM/normoalbuminuria, 59.4 ± 8.5 yr, M/F	Tehran	Cross sectional	PCR	Positive significant association between DD carriers and progression of albuminuria (*P* < 0.01) but not its development
Nikzamir et al., 2006 [105]	ACE/*I/D *	85 T2DM/DN, 85 T2DM/non-DN, and 91 healthy, 37–67 yr, M/F	Tehran	Case control	PCR	Positive significant association between DD carriers and T2DM (*P* = 0.006) but not DN

T2DM patients and retinopathy						
Abbasi et al., 2013 [106]	GSTM1/*null/positive *	80 DR and 80 healthy, 30–70 yr, M/F	Rasht	Case control	ARMS-PCR	Null genotype significant association, *P* < 0.05
Dadbinpour et al., 2013 [107]	(i) GSTM1/*null/positive* (ii) GSTT1/*null/positive *	57 DR and 58 non-DR, 35–65 yr, M/F	Yazd	Case control	Multiplex PCR	(i) Null genotype of GSTM1 or GSTT1 significant association (*P* = 0.04) (ii) Nonsignificant association of GSTT1 null carriers with DR
Feghhi et al., 2011 [108]	VEGF/*+405G/C *	119 diabetics with PDR and 279 diabetics with NPDR, 47–66 yr, M/F	Ahvaz	Case control	PCR-RFLP	Positive significant association between GG carriers and diabetic retinopathy (*P* = 0.005)

T2DM patients and foot ulcer						
Amoli et al., 2011 [109]	(i) VEGF/*−7C/T* (ii) VEGF/*−2578C/A *	247 T2DM with DFU, 241 T2DM without DFU, and 98 healthy, 43–64, M/F	Tehran	Case control	ARMS-PCR	(i) Nonsignificant association of −7C/T carriers with DFU (ii) Positive significant association between AA carriers and DFU (*P* = 0.03)

SNP, single nucleotide polymorphism; T1DM, type 1 diabetes mellitus; CTLA-4, cytotoxic T lymphocyte associated antigen 4; T2DM, type 2 diabetes mellitus; PCR, polymerase chain reaction; RFLP, restriction fragment length polymorphism; IL, interleukin; SS-PCR, sequence specific PCR; VDR, vitamin D receptor; TAP2, transporter 2 ATP-binding cassette; ARMS-PCR, amplification refractory mutation system PCR; PCR-SSP, PCR single specific primer; TGF*β*, transforming growth factor *β*; DM, diabetes mellitus; CTLA-4, cytotoxic T lymphocyte associated antigen 4; MODY, maturity onset diabetes of the young; HNF-1*α*, hepatocyte nuclear factor 1*α*; MVC, microvascular complications; TCF7L2, transcription factor 7-like 2; PCR-SSCA, PCR single strand conformation polymorphism analysis; ER*α*, estrogen receptor *α*; ND, nondiabetics; TC, total cholesterol; TG, triglycerides; PPAR-*γ*2, peroxisome proliferator-activates receptor *γ*; GLP-1R, glucagon-like peptid 1 receptor; APO, apolipoprotein; CXCL5, chemokine C-X-C motif ligand 5; SDF-1*β*, stromal derived factor-1*β*; IRS1 & 2, insulin receptor substrate 1 & 2; GST, glutathione-S-transferase; SLC30A8, soluble carrier 30 A8; KCNJ, Potassium inwardly-rectifying channel; eNOS, endothelial nitric oxide synthase; VNTR, variable number of tandem repeats; ENPP1, ectoenzyme nucleotide pyrophosphate phosphodiesterase 1; SUMO, small ubiquitin-like modifier 4; UCP2, uncoupling protein 2; MTHFR, methylenetetrahydrofolate reductase; IFN*γ*, interferon *γ*; CCR5, C-C chemokine receptor type 5; INSR, insulin receptor; CSGE, conformation-sensitive gel electrophoresis; ACE, angiotensin I converting enzyme; HFE, hemochromatosis gene; PTPN1, protein tyrosin Phosphatase 1B; CAD, coronary artery disease; CETP, cholesteryl ester transfer protein; AT1R, angiotensin I receptor; MMP3, matrix metalloproteinase 3; CAS, coronary artery stenosis; BChE K, Butyrylcholinesterase K; DN, diabetic nephropathy; AT2R, angiotensin II receptor; VEGF, vascular epithelium growth factor; DR, diabetic retinopathy; PDR, proliferative diabetic retinopathy; NPDR, non-PDR; DFU, diabetic foot ulcer.

## References

[1] American Diabetes Association (2004). Diagnosis and classification of diabetes mellitus. *Diabetes Care*.

[2] World Health Organization (WHO) (1965). *Diabetes Mellitus, Report of a WHO Expert Committee*.

[3] World Health Organization (WHO) (1980). WHO expert committee on diabetes mellitus. *WHO Technical Report Series No.*.

[4] International Diabetes Federation (2013). *IDF Diabetes Atlas*.

[5] Najafipoor F., Azizi F., Zareizadeh M. (2004). Epidemiologic investigation of familial type 2 diabetes in Tehran. *Iranian Journal of Diabetes and Lipid Disorders*.

[6] IDF Diabetes Atlas (2012). *New Estimates for 2012 of Diabetes Prevalence, Mortality, and Healthcare Expenditures*.

[7] Haghdoost A. A., Rezazadeh-Kermani M., Sadghirad B., Baradaran H. R. (2009). Prevalence of type 2 diabetes in the Islamic Republic of Iran: systematic review and meta-analysis. *Eastern Mediterranean Health Journal*.

[8] Akrami S. M. (2007). Genetic counseling in diabetes. *Iranian Journal of Diabetes and Lipid Disorders*.

[9] National Diabetes Data Group (1979). Classification and diagnosis of diabetes mellitus and other categories of glucose intolerance. *Diabetes*.

[10] Xiang K., Wang Y., Zheng T. (2004). Genome-wide search for type 2 diabetes/impaired glucose homeostasis susceptibility genes in the Chinese. *Diabetes*.

[11] Luo T. H., Zhao Y., Li G. (2001). A genome-wide search for type II diabetes susceptibility genes in Chinese Hans. *Diabetologia*.

[12] Ali O. (2013). Genetics of type 2 diabetes. *World Journal of Diabetes*.

[13] Prokopenko I., McCarthy M. I., Lindgren C. M. (2008). Type 2 diabetes: new genes, new understanding. *Trends in Genetics*.

[14] Abbas S., Raza S. T., Ahmed F., Ahmad A., Rizvi S., Mahdi F. (2013). Association of genetic polymorphism of PPAR*γ*-2, ACE, MTHFR, FABP-2 and FTO genes in risk prediction of type 2 diabetes mellitus. *Journal of Biomedical Science*.

[15] Huang Q.-Y., Cheng M.-R., Ji S.-L. (2006). Linkage and association studies of the susceptibility genes for type 2 diabetes. *Acta Genetica Sinica*.

[16] Li S., Shin H. J., Ding E. L., van Dam R. M. (2009). Adiponectin levels and risk of type 2 diabetes: a systematic review and meta-analysis. *The Journal of the American Medical Association*.

[17] Bakhtiari S., Babakhani A., Maleki M. H. (2012). Investigation of the association between rs2970847 polymorphism of PGC-1*α* gene and type 2 diabetes mellitus, a systematic review and meta analysis. *Journal of Research of Medical School of Shahid Beheshti University*.

[18] Ounissi-Benkalha H., Polychronakos C. (2008). The molecular genetics of type 1 diabetes: new genes and emerging mechanisms. *Trends in Molecular Medicine*.

[19] Moher D., Liberati A., Tetzlaff J., Altman D. G. (2009). Preferred reporting items for systematic reviews and meta-analyses: the PRISMA statement. *British Medical Journal*.

[20] World Health Organization (WHO) (2006). Definition and diagnosis of diabetes mellitusand intermediate hyperglycemia. *Report of a WHO/IDF Consultation*.

[21] Vandenbroucke J. P., von Elm E., Altman D. G. (2007). Strengthening the reporting of observational studies in epidemiology (STROBE): explanation and elaboration. *PLoS Medicine*.

[22] Ahmadi S., Rostamzadeh J., Khosravi D., Shariati P., Shakiba N. (2013). Association of CTLA-4 gene 49A/G polymorphism with the incidence of type 1 diabetes mellitus in the Iranian Kurdish population. *Pakistan Journal of Biological Sciences*.

[23] Karamizadeh Z., Kamali Sarvestani E., Saki F. (2013). Investigation of osteopontin levels and genomic variation of osteopontin and its receptors in type 1 diabetes mellitus. *Journal of Endocrinological Investigation*.

[24] Rahbani-Nabar M., Rezaie A., Bazzaz S., Zadeh N. A. (2013). Association of 137 polymorphism in the interleukin-18 gene promoter with diabetes melhitus type 1 in East Azerbaijan population. *Medical Journal of Tabriz University of Medical Sciences and Health Services*.

[25] Bonakdaran S., Abbaszadegan M. R., Dadkhah E., Khajeh-Dalouie M. (2012). Vitamin D receptor gene polymorphisms in type 1 diabetes mellitus: a new pattern from Khorasan province, Islamic Republic of Iran. *Eastern Mediterranean Health Journal*.

[26] Mohammadnejad Z., Ghanbari M., Ganjali R. (2012). Association between vitamin D receptor gene polymorphisms and type 1 diabetes mellitus in Iranian population. *Molecular Biology Reports*.

[27] Afshari J. T., Taghavi S. M., Fatemi S. S., Rafatpanah H., Gheybi F., Nezamdoost N. (2011). TAP2 polymorphisms in Iranian patients with type I diabetes mellitus. *International Journal of Genetics and Molecular Biology*.

[28] Massoud A., Sheikh Bahai N., Massoud M. (2009). IL18 gene polymorphism in type I diabetic patients: a case-control study. *Tehran University Medical Journal*.

[29] Masood M., Salehi I., Sheykh Bahayi N., Vojgani M., Rajab A. A., Massoud A. (2007). Investigation of codon 25 polymorphism of TGF*β* gene in type I diabetes mellitus patients. *Journal of Medical Faculty Guilan University of Medical Sciences*.

[30] Masoud A. H., Keihani A., Sheykhbahaee N. (2007). Investigation of polymorphism of IL-12 in type I diabetes mellitus patients. *Medical Journal of Mashhad University of Medical Sciences*.

[31] Mojtahedi Z., Naeimi S., Farjadian S., Omrani G. R., Ghaderi A. (2006). Association of IL-18 promoter polymorphisms with predisposition to Type 1 diabetes. *Diabetic Medicine*.

[32] Mojtahedi Z., Omrani G. R., Doroudchi M., Ghaderi A. (2005). CTLA-4 +49 A/G polymorphism is associated with predisposition to type 1 diabetes in Iranians. *Diabetes Research and Clinical Practice*.

[33] Zamani M., Tabatabaiefar M. A., Esfahani A. S., Mostafavi F., Sotoudeh A., Larijani B. (2005). Correlation between the CD4 gene polymorphism with type 1 diabetes mellitus in the Iranian population. *Iranian Journal of Diabetes and Lipid Disorders*.

[34] Taghavi S. M., Fatemi S. S., Rafatpanah H., Ganjali R., Tavakolafshari J., Valizadeh N. (2009). Mutations in the coding regions of the hepatocyte nuclear factor 4 alpha in Iranian families with maturity onset diabetes of the young. *Cardiovascular Diabetology*.

[35] Amiri A. A., Hashemi-Soteh M. B., Haghshenas M. R., Daneshvar F., Rastegar A., Farazmand T. (2013). Haptoglobin polymorphism in individuals with type 2 diabetic microangiopathy. *North American Journal of Medical Sciences*.

[36] Andalib S., Vaseghi G., Motavallian A. (2013). Association of polymorphism of ser311cys paraoxonase-2 gene with type 2 diabetes mellitus in Iran. *International Journal of Preventive Medicine*.

[37] Kohan L., Nasiri M., Habib A., Bolhasani A. (2013). Association of G-2548A polymorphism in the promoter of leptin gene with plasma leptin level and risk of type 2 diabetes. *Journal of Shahid Sadoughi University of Medical Sciences*.

[38] Alami F. M., Samaei N. M., Ahmadi M. (2013). Association of transcription factor 7-like 2 (*Tcf7l2*) gene haplotypes with the risk of type 2 diabetes mellitus in Iran. *Advances in Biological Research*.

[39] Mahmazi S., Parivar K., Rahnema M., Ohadi M. (2013). Calreticulin novel mutations in type 2 diabetes mellitus. *International Journal of Diabetes in Developing Countries*.

[40] Mohammadi F., Pourahmadi M., Mosalanejad M., Jamali H., Ghobadifar M. A., Erfanian S. (2013). Association of estrogen receptor *α* genes *Pvu*II and *Xba*I polymorphisms with type 2 diabetes mellitus in the inpatient population of a hospital in Southern Iran. *Diabetes and Metabolism Journal*.

[41] Motavallian A., Andalib S., Vaseghi G., Mirmohammad-Sadeghi H., Amini M. (2013). Association between PRO12ALA polymorphism of the PPAR-*γ*2 gene and type 2 diabetes mellitus in Iranian patients. *Indian Journal of Human Genetics*.

[42] Sepahi S., Jalal R., Toluinia B., Asoodeh A., Darvish J. (2013). Evaluation of relationship between HNF-1*α* and GLP-1R polymorphisms and type 2 diabetes in a population living in northeast of Iran. *Journal of Cell and Molecular Research*.

[43] Sheikhha M. H., Afkhami-Ardekani M., Mirjalili S. M. R., Dehghani S. M. R., Ghadimi H. R. (2013). Investigating the frequency of MSPI polymorphism of APOA1 gene in type II diabetic patients and comparing it with this frequency in nondiabetics. *Genetics in the 3rd Millennium*.

[44] Yaghoubi H., Haghi M., Solhi S. (2013). Detection of CXCL5 gene polymorphism with diabetes in Ardabil province. *Journal of Ardabil University of Medical Sciences*.

[45] Bahreini F., Ardebili S. M. M., Farajnia S. (2012). A study on association of SNP-43 polymorphism in Calpain-10 gene with type 2 diabetes mellitus in the population of Eastern Azerbaijan province. *Iranian South Medical of Journal*.

[46] Derakhshan R., Arababadi M. K., Ahmadi Z. (2012). Increased circulating levels of SDF-1 (CXCL12) in type 2 diabetic patients are correlated to disease state but are unrelated to polymorphism of the SDF-1*β* gene in the Iranian population. *Inflammation*.

[47] Haghani K., Bakhtiyari S. (2012). The study on the relationship between IRS-1 Gly972Arg and IRS-2 Gly1057Asp polymorphisms and type 2 diabetes in the Kurdish ethnic group in west Iran. *Genetic Testing and Molecular Biomarkers*.

[48] Alami F. M., Ahmadi M., Bazrafshan H. (2012). Association of the TCF7L2 rs12255372 (G/T) variant with type 2 diabetes mellitus in an Iranian population. *Genetics and Molecular Biology*.

[49] Meshkani R., Saberi H., Mohammadtaghvaei N., Tabatabaiefar M. A. (2012). Estrogen receptor alpha gene polymorphisms are associated with type 2 diabetes and fasting glucose in male subjects. *Molecular and Cellular Biochemistry*.

[50] Moasser E., Kazemi-Nezhad S. R., Saadat M., Azarpira N. (2012). Study of the association between glutathione *S*-transferase (GSTM1, GSTT1, GSTP1) polymorphisms with type II diabetes mellitus in southern of Iran. *Molecular Biology Reports*.

[51] Mohaddes S. M., Karami F., Gharesouran J., Bahrami A. (2012). The soluble carrier 30 A8 (SLC30A8) gene polymorphism and risk of Diabetes Mellitus Type 2 in Eastern Azerbijan population of Iran. *Journal of Sciences, Islamic Republic of Iran*.

[52] Oladi M. R., Behravan J., Hassani M. (2012). Glucokinase gene promoter-30G>A polymorphism: a cross-sectional association study with obesity, diabetes Mellitus, hyperlipidemia, hypertension and metabolic syndrome in an Iranian hospital. *Revista de Nutricao*.

[53] Palizban A., Nikpour M., Salehi R., Maracy M.-R. (2012). Association of a common variant in TCF7L2 gene with type 2 diabetes mellitus in a Persian population. *Clinical and Experimental Medicine*.

[54] Tabatabaei-Malazy O., Fakhrzadeh H., Qorbani M. (2012). Apolipoprotein E gene polymorphism and its effect on anthropometric measures in normoglycemic subjects and type 2 diabetes. *Journal of Diabetes & Metabolic Disorders*.

[55] Ghasemi M., Habibipour R., Keshavarz P. (2012). The association study of the E23k Kcnj11 variant with progression of type 2 diabetes among obese individuals in a population in the North of Iran. *Iranian Journal of Endocrinology and Metabolism*.

[56] Ranjbar S. H., Amoli M. M., Sajadi M. (2011). Genetic association analysis of the adiponectin polymorphisms in type 2 diabetes with and without complications. *Iranian Journal of Diabetes and Lipid Disorders*.

[57] Mehrab-Mohseni M., Tabatabaei-Malazy O., Hasani-Ranjbar S. (2011). Endothelial nitric oxide synthase VNTR (intron 4 a/b) polymorphism association with type 2 diabetes and its chronic complications. *Diabetes Research and Clinical Practice*.

[58] Nosratabadi R., Arababadi M. K., Salehabad V. A. (2011). Vitamin D receptor polymorphisms in type 2 diabetes in southeastern Iranian patients. *Laboratory Medicine*.

[59] Saberi H., Mohammadtaghvaei N., Gulkho S. (2011). The ENPP1 K121Q polymorphism is not associated with type 2 diabetes and related metabolic traits in an Iranian population. *Molecular and Cellular Biochemistry*.

[60] Fallah S., Jafarzadeh M., Hedayati M. (2010). No association of the SUMO4 polymorphism M55V variant in type 2 diabetes in Iranian subjects. *Diabetes Research and Clinical Practice*.

[61] Heidari J., Akrami S. M., Heshmat R., Amiri P., Fakhrzadeh H., Pajouhi M. (2010). Association study of the −866G/A UCP2 gene promoter polymorphism with type 2 diabetes and obesity in a tehran population: a case control study. *Archives of Iranian Medicine*.

[62] Nazem H., Takhshid M. A., Tabei S. M. B., Sholevar F., Entezam M., Manoochehri J. (2010). Investigation of association between serotonin transporter gene and type 2 diabetes mellitus. *Iranian Journal of Diabetes and Lipid Disorders*.

[63] Bazzaz J. T., Shojapoor M., Nazem H. (2010). Methylenetetrahydrofolate reductase gene polymorphism in diabetes and obesity. *Molecular Biology Reports*.

[64] Emamgholipour S., Hossein-nezhad A., Najmafshar A., Rahmani M., Larijani B. (2009). Promoter resistin gene polymorphism in patients with type 2 diabetes and its influence on concerned metabolic phenotypes. *Iranian Journal of Diabetes and Lipid Disorders*.

[65] Hasani-Ranjbar S., Amiri P., Namakchian M. (2009). Investigation of association between *CXCL5* gene polymorphism and diabetes. *Iranian Journal of Diabetes and Lipid Disorders*.

[66] Kazemi Arababadi M., Pourfathollah A. A., Daneshmandi S. (2009). Evaluation of relation between IL-4 and IFN-*γ* polymorphisms and type 2 diabetes. *Iranian Journal of Basic Medical Sciences*.

[67] Arababadi M. K., Naghavi N., Hassanshahi G., Sajadi M. (2009). Evaluating the association of CCR5-d32 mutation with type 2 diabetes in Rafsanjanese patients. *Feyz Journal of Kashan University of Medical Sciences*.

[68] Kazemi B., Seyed N., Moslemi E. (2009). Insulin receptor gene mutations in Iranian patients with type II diabetes mellitus. *Iranian Biomedical Journal*.

[69] Mirzaei H., Akrami S. M., Golmohammadi T. (2009). Polymorphism of Pro12Ala in the peroxisome proliferator-activated receptor 2 gene in Iranian diabetic and obese subjects. *Metabolic Syndrome and Related Disorders*.

[70] Nikzamir A., Nakhjavani M., Golmohammadi T., Dibai L., Saffary R. (2008). Polymorphism in the angiotensin-converting enzyme (ACE) gene and ACE activity in type 2 diabetic patients. *Acta Medica Iranica*.

[71] Sharifi F., Esmaeilzadeh A., Zali M. (2008). Hemochromatosis gene (HFE) mutations in patients with type 2 diabetes and their control group in an Iranian population. *Saudi Medical Journal*.

[72] Besharati A., Akrami S. M., Heshmat R., Yaghmaee P., Alirezapoor B. (2007). Investigation of association between apoAI gene polymorphisms and lipid profiles and type 2 diabetes mellitus in Iranian population. *Journal of Islamic Republic of Iran Medical Council*.

[73] Hasani-Ranjbar S., Tavakkoly Bazzaz J., Amiri P., Amoli M. M., Larijani B. (2007). Investigation of the frequency of +45T/G polymorphism of adiponectin gene in type 2 diabetic patients in a Tehranian population. *Iranian Journal of Diabetes and Lipid Disorders*.

[74] Meshkani R., Taghikhani M., Al-Kateb H. (2007). Polymorphisms within the protein tyrosine phosphatase IB (*PTPN1*) gene promoter: functional characterization and association with type 2 diabetes and related metabolic traits. *Clinical Chemistry*.

[75] Meshkani R., Taghikhani M., Larijani B. (2007). Pro12Ala polymorphism of the peroxisome proliferator-activated receptor-*γ*2 (PPAR*γ*-2) gene is associated with greater insulin sensitivity and decreased risk of type 2 diabetes in an Iranian population. *Clinical Chemistry and Laboratory Medicine*.

[76] Namvaran F., Rahimi M.-P., Azarpira N., Dabbaghmanesh M. H. (2012). Polymorphism of adiponectin (45T/G) and adiponectin receptor-2 (795G/A) in an iranian population: relation with insulin resistance and response to treatment with pioglitazone in patients with type 2 diabetes mellitus. *Molecular Biology Reports*.

[77] Namvaran F., Azarpira N., Rahimi-Moghaddam P., Dabbaghmanesh M. H. (2011). Polymorphism of peroxisome proliferator-activated receptor *γ* (PPAR*γ*) Pro12Ala in the Iranian population: Relation with insulin resistance and response to treatment with pioglitazone in type 2 diabetes. *European Journal of Pharmacology*.

[78] Hossein-nezhad A., Mirzaei K., Shabani P. (2009). Association of VDR gene polymorphism with insulin resistance in diabetic patient's running title: VDR and insulin resistance. *Iranian Journal of Diabetes and Lipid Disorders*.

[79] Moosapoor A., Taghikhani M., Meshkani R., Khatami S., Bakhtiari S., Haghani K. (2007). Association of 3′UTR (1484insG) polymorphism of PTP1B gene with Type 2 diabetes, in-sulin resistance and obesity in a Tehranian population. *Journal of School of Public Health and Institute of Public Health Research*.

[80] Bayatmakoo R., Mobaiyen H., Monfaredan A., Sofiani K. B., Benissy R. (2013). The study of effects common paraoxonase polymorphism (L55M) on atherosclerosis risk in diabetic patients by PCR-RFLP. *European Journal of Experimental Biology*.

[81] Bayatmakoo R., Monfaredan A., Bargahi N., Mobaiyen H., Aslanabadi N. (2012). Paraoxonase gene polymorphism and atherosclerosis risk in diabetic patients. *Journal of Azad University of Medical Sciences*.

[82] Esteghamati A., Mansournia N., Nakhjavani M., Mansournia M. A., Nikzamir A., Abbasi M. (2012). Association of +45(T/G) and +276(G/T) polymorphisms in the adiponectin gene with coronary artery disease in a population of Iranian patients with type 2 diabetes. *Molecular Biology Reports*.

[83] Rahimi Z., Nourozi-Rad R., Parsian A. (2012). Strong interaction between T allele of endothelial nitric oxide synthase with B1 allele of cholesteryl ester transfer protein TaqIB highly elevates the risk of coronary artery disease and type 2 diabetes mellitus. *Human Genomics*.

[84] Assali A., Ghayour-Mobarhan M., Sahebkar A. (2011). Association of angiotensin II type 1 receptor gene A1166C polymorphism with the presence of diabetes mellitus and metabolic syndrome in patients with documented coronary artery disease. *European Journal of Internal Medicine*.

[85] Emamgholipour S., Hossein-nezhad A., Mohajerani S. A., Shirzad M., Larijani B. (2009). Association between promoter resistin gene polymorphism and coronary artery disease in diabetic and non diabetic patients. *Iranian Journal of Diabetes and Lipid Disorders*.

[86] Fallah S., Seifi M., Samadikuchaksaraei A. (2010). Risk of coronary artery stenosis in Iranian type 2 diabetics: is there a role for matrix metalloproteinase-3 gene (-1612 5A/6A) polymorphism?. *Journal of Physiology and Biochemistry*.

[87] Vaisi-Raygani A., Rahimi Z., Tavilani H., Pourmotabbed T. (2010). Butyrylcholinesterase K variant and the APOE-epsilon 4 allele work in synergy to increase the risk of coronary artery disease especially in diabetic patients. *Molecular Biology Reports*.

[88] Rahimi Z., Nomani H., Mozafari H. (2009). Factor v G1691A, prothrombin G20210A and methylenetetrahydrofolate reductase polymorphism C677T are not associated with coronary artery disease and type 2 diabetes mellitus in western Iran. *Blood Coagulation & Fibrinolysis*.

[89] Nakhjavani M., Esfahanian F., Jahanshahi A. (2007). The relationship between the insertion/deletion polymorphism of the ACE gene and hypertension in Iranian patients with type 2 diabetes. *Nephrology Dialysis Transplantation*.

[90] Vaisi-Raygani A., Rahimi Z., Nomani H., Tavilani H., Pourmotabbed T. (2007). The presence of apolipoprotein *ε*4 and *ε*2 alleles augments the risk of coronary artery disease in type 2 diabetic patients. *Clinical Biochemistry*.

[91] Rahimi Z., Shahvaisi-Zadeh F., Sadeghei S., Vessal M., Yavari N. (2013). eNOS 4a/b polymorphism and its interaction with eNOS G894T variants in type 2 diabetes mellitus: modifying the risk of diabetic nephropathy. *Disease Markers*.

[92] Rahimi Z., Mansouri Zaveleh O., Rahimi Z., Abbasi A. (2013). AT2R -1332 G:A polymorphism and diabetic nephropathy in type 2 diabetes mellitus patients. *Journal of Renal Injury Prevention*.

[93] Shahsavar F., Kheirollahi A., Jafarzadeh M., hedayati M. (2013). SUMO4 M55V Polymorphism is associated with diabetic nephropathy in Iranian type 2 diabetes patients. *Life Science Journal*.

[94] Arababadi M. K., Mirzaei M. R., Sajadi S. M. A. (2012). Interleukin (IL)-10 gene polymorphisms are associated with type 2 diabetes with and without nephropathy: a study of patients from the Southeast Region of Iran. *Inflammation*.

[95] Nikzamir A., Esteghamati A., Hammedian A. A., Mahmoudi T. (2012). The role of vascular endothelial growth factor +405 G/C polymorphism and albuminuria in patients with type 2 diabetes mellitus. *Molecular Biology Reports*.

[96] Rahimi Z., Hasanvand A., Felehgari V. (2012). Interaction of MTHFR 1298C with ACE D allele augments the risk of diabetic nephropathy in Western Iran. *DNA and Cell Biology*.

[97] Rahimi Z., Vaisi-Raygani A., Parsian A. (2012). Concomitant presence of endothelial nitric oxide 894T and angiotensin II-converting enzyme D alleles are associated with diabetic nephropathy in a Kurdish population from Western Iran. *Nephrology (Carlton, Vic)*.

[98] Felehgari V., Rahimi Z., Mozafari H., Vaisi-Raygani A. (2011). ACE gene polymorphism and serum ACE activity in Iranians type II diabetic patients with macroalbuminuria. *Molecular and Cellular Biochemistry*.

[99] Jafari Y., Rahimi Z., Vaisi-Raygani A., Rezaei M. (2011). Interaction of eNOS polymorphism with MTHFR variants increase the risk of diabetic nephropathy and its progression in type 2 diabetes mellitus patients. *Molecular and Cellular Biochemistry*.

[100] Rahimi Z., Felehgari V., Rahimi M. (2011). The frequency of factor V Leiden mutation, ACE gene polymorphism, serum ACE activity and response to ACE inhibitor and angiotensin II receptor antagonist drugs in Iranians type II diabetic patients with microalbuminuria. *Molecular Biology Reports*.

[101] Arababadi M. K. (2010). Interleukin-4 gene polymorphisms in type 2 diabetic patients with nephropathy. *Iranian Journal of Kidney Diseases*.

[102] Nosratabadi R., Arababadi M. K., Salehabad V. A. (2010). Polymorphisms within exon 9 but not intron 8 of the vitamin D receptor are associated with the nephropathic complication of type-2 diabetes. *International Journal of Immunogenetics*.

[103] Rahimi M., Hasanvand A., Rahimi Z. (2010). Synergistic effects of the MTHFR C677T and A1298C polymorphisms on the increased risk of micro- and macro-albuminuria and progression of diabetic nephropathy among Iranians with type 2 diabetes mellitus. *Clinical Biochemistry*.

[104] Nikzamir A., Esteghamati A., Feghhi M., Nakhjavani M., Rashidi A., Reza J. Z. (2009). The insertion/deletion polymorphism of the angiotensin-converting enzyme gene is associated with progression, but not development, of albuminuria in Iranian patients with type 2 diabetes. *Journal of the Renin-Angiotensin-Aldosterone System*.

[105] Nikzamir A. R., Golmohammadi T., Nakhjavani M., Zahraei M., Amirzargar A. A. (2006). Angiotensin converting enzyme gene polymorphism in Iranian patients with type 2 diabetes. *Iranian Journal of Immunology*.

[106] Abbasi N., Salehi Z., Alizadeh Y. (2013). Analysis of glutathione S-transferase M1 (GSTM1) deletion in diabetic retinopathy. *Journal of Guilan University of Medical Sciences*.

[107] Dadbinpour A., Sheikhha M., Darbouy M., Afkhami-Ardekani M. (2013). Investigating GSTT1 and GSTM1 null genotype as the risk factor of diabetes type 2 retinopathy. *Journal of Diabetes & Metabolic Disorders*.

[108] Feghhi M., Nikzamir A., Esteghamati A., Mahmoudi T., Yekaninejad M. S. (2011). Relationship of vascular endothelial growth factor (VEGF) +405 G/C polymorphism and proliferative retinopathy in patients with type 2 diabetes. *Translational Research*.

[109] Amoli M. M., Hasani-Ranjbar S., Roohipour N. (2011). VEGF gene polymorphism association with diabetic foot ulcer. *Diabetes Research and Clinical Practice*.

[110] Chu H., Wang M., Zhong D. (2013). AdipoQ polymorphisms are associated with type 2 diabetes mellitus: a meta-analysis study. *Diabetes/Metabolism Research and Reviews*.

[111] Wang J., Zhang J., Li L. (2013). Association of rs12255372 in the *TCF7L2* gene with type 2 diabetes mellitus: a meta-analysis. *Brazilian Journal of Medical and Biological Research*.

[112] Saadat M. (2013). Null genotypes of glutathione S-transferase M1 (GSTM1) and T1 (GSTT1) polymorphisms increased susceptibility to type 2 diabetes mellitus, a meta-analysis. *Gene*.

[113] Tang S.-T., Wang C.-J., Tang H.-Q., Zhang Q., Wang Y. (2013). Evaluation of glutathione S-transferase genetic variants affecting type 2 diabetes susceptibility: a meta-analysis. *Gene*.

[114] Zhong J.-H., Rodríguez A. C., Yang N.-N., Li L.-Q. (2013). Methylenetetrahydrofolate reductase gene polymorphism and risk of type 2 diabetes mellitus. *PLoS ONE*.

[115] Yang S., Zhang J., Feng C., Huang G. (2013). MTHFR 677 T variant contributes to diabetic nephropathy risk in Caucasian individuals with type 2 diabetes: a meta-analysis. *Metabolism: Clinical and Experimental*.

[116] Hua Y., Shen J., Song Y., Xing Y., Ye X. (2013). Interleukin-10 −2592C/A, −2819C/T and −21082A/G polymorphisms with risk of type 2 diabetes mellitus: a HuGE review and meta-analysis. *PLoS ONE*.

[117] Helaly M. A.-H., Hatata E.-S. Z., Abu-Elmagd M. (2013). Association of IL-10 and IL-6 gene polymorphisms with type 2 diabetes mellitus among Egyptian patients. *European Journal of General Medicine*.

[118] Vats S., Sambyal V., Bhanwer A. J. S. (2013). Genetic links between coronary artery disease and type 2 diabetes. *Human Biology Review*.

[119] Wang G., Zhang Q., Xu N. (2014). Associations between two polymorphisms (FokI and BsmI) of vitamin D receptor gene and type 1 diabetes mellitus in Asian population: a meta-analysis. *PLoS ONE*.

[120] Tizaoui K., Kaabachi W., Hamzaoui A., Hamzaoui K. (2014). Contribution of VDR polymorphisms to type 1 diabetes susceptibility: systematic review of case-control studies and meta-analysis. *Journal of Steroid Biochemistry and Molecular Biology*.

[121] Sun L., Yuan Q., Cao N. (2014). *VEGF* genetic polymorphisms may contribute to the risk of diabetic nephropathy in patients with diabetes mellitus: a meta-analysis. *The Scientific World Journal*.

[122] Dellamea B. S., Pinto L. C. F., Leitão C. B., Santos K. G., Canani L. H. S. (2014). Endothelial nitric oxide synthase gene polymorphisms and risk of diabetic nephropathy: a systematic review and meta-analysis. *BMC Medical Genetics*.

[123] Ma Z. J., Chen R., Ren H.-Z., Guo X., Chen J. G., Chen L.-M. (2014). Endothelial nitric oxide synthase (eNOS) 4b/a polymorphism and the risk of diabetic nephropathy in type 2 diabetes mellitus: a meta-analysis. *Meta Gene*.

[124] Ehm M. G., Karnoub M. C., Sakul H. (2000). Genomewide search for type 2 diabetes susceptibility genes in four american population. *The American Journal of Human Genetics*.

[125] Busfield F., Duffy D. L., Kesting J. B. (2002). A genomewide search for type 2 diabetes-susceptibility genes in indigenous Australians. *American Journal of Human Genetics*.

[126] Elbein S. C., Hoffman M. D., Teng K., Leppert M. F., Hasstedt S. J. (1999). A genome-wide search for type 2 diabetes susceptibility genes in Utah Caucasians. *Diabetes*.

[127] Rotimi C. N., Chen G., Adeyemo A. A. (2004). A genome-wide search for type 2 diabetes susceptibility genes in West Africans. *Diabetes*.

[128] Cauchi S., Meyre D., Durand E. (2008). Post genome-wide association studies of novel genes associated with type 2 diabetes show gene-gene interaction and high predictive value. *PLoS ONE*.

